# Left Atrial Strain as Evaluated by Two-Dimensional Speckle Tracking Predicts Left Atrial Appendage Dysfunction in Chinese Patients with Atrial Fibrillation

**DOI:** 10.1155/2020/5867617

**Published:** 2020-03-21

**Authors:** Yu Wang, Mingqi Li, Lishan Zhong, Siqi Ren, Hezhi Li, Yongwen Tang, Zhilian Li, Hongwen Fei

**Affiliations:** ^1^Department of Cardiology, Guangdong Cardiovascular Institute, Guangdong Provincial People's Hospital, Guangdong Academy of Medical Sciences, Guangzhou, Guangdong 510100, China; ^2^Shantou University Medical College, Shantou, Guangdong 515000, China; ^3^Department of Medical Imaging, Guangzhou First People's Hospital, School of Medicine, South China University of Technology, Guangzhou, Guangdong 515000, China

## Abstract

Left atrial appendage (LAA) dysfunction identified by transesophageal echocardiography (TEE) is a powerful predictor of stroke in patients with atrial fibrillation (AF). The aim of our study is to assess if there is a correlation between the left atrial (LA) functional parameter and LAA dysfunction in the AF patients. This cross-sectional study included a total of 249 Chinese AF patients who did not have cardiac valvular diseases and were undergoing cardiac ablation. TEE was performed in all the patients who were categorized into two groups according to their left atrial appendage (LAA) function. A total of 120 of the 249 AF patients had LAA dysfunction. Univariate and multivariate logistic regression was conducted to assess the independent factors that correlated with the LAA dysfunction. Different predictive models for the LAA dysfunction were compared with the receiver operating characteristic (ROC) curve. The final ROC curve on the development and validation datasets was drawn based on the calculation of each area under the curves (AUC). Univariate and multivariate analysis showed that the peak left atrial strain (PLAS) was the most significant factor that correlated with the LAA dysfunction. PLAS did not show inferiority amongst all the models and revealed strong discrimination ability on both the development and validation datasets with AUC 0.818 and 0.817. Our study showed that a decrease in PLAS is independently associated with LAA dysfunction in the AF patients.

## 1. Introduction

AF is the most frequent type of arrhythmia, having a global prevalence of 1% to 2%. AF is a public health challenge associated with high comorbidity and an increased mortality risk [1–4]. For example, AF increases the risk of stroke by five times and is observed in ∼33.3% of all ischemic stroke patients [5, 6]. In patients presenting with nonvalvular AF, over 90% of the thrombi originate in the LAA. Anatomical remodeling, atrial fibrosis, and a decline in the contractility of the LA myocardium are factors associated with left atrial thrombus/spontaneous echocardiographic contrast (LAT/SEC), as well as reductions in LAA flow velocities by TEE, which has been found to predispose patients to stroke [7]. Conventionally, TTE has been used to observe anatomical changes of LA following remodeling in the AF patients [8]; however, it has been increasingly replaced with two-dimensional (2D) speckle tracking echocardiography in order to evaluate LA function [9–11]. Most studies that report the association between LA and LAA function [12–15] were carried out on occidental populations. Compared to Western countries, stroke incidence in China is universally higher and the number of cases undergoing AF ablation procedures has been rapidly increasing. Moreover, stroke, which is associated with AF, is the second leading cause of death in China [16]. Here, we aim to provide a noninvasive imaging modality to evaluate the LAA function in Chinese patients.

## 2. Methods

**Figure 1:**
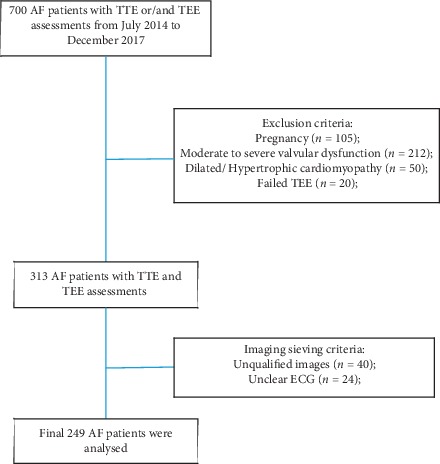
Flow chart of the study screening and selection process for patients used in the study.

A retrospective and cross-sectional study was conducted at the Guangdong Provincial People's Hospital. This study has been approved by the institutional review board of the hospital. A total of 700 patients diagnosed with AF at the Guangdong Provincial People's Hospital from July 2014 to December 2017 (Figure 1) were included in this study. Patients exhibiting the following criteria were excluded from this study: pregnancy (*n* = 105), moderate to severe valvular dysfunction (*n* = 212), dilated/hypertrophic cardiomyopathy (*n* = 50), failed TEE (*n* = 20), and cases containing unqualified images (*n* = 64), either through failure to capture the total LA geometry (mostly incomplete contours of the LA roof) or image quality precluding speckle tracking. The patients were informed of research objectives and protocols, and they provided consent to participate in the study. Clinical information was collected at the time of their first hospital visit, including demographic data, current medications, and past medical history (PMH). The CHA2DS2-VASc score was calculated for each patient, and they underwent evaluation with TTE or/and TEE before proceeding to AF management.

### 2.1. Transthoracic Echocardiography

A two-dimension gray scale echocardiography was used to obtain standard view images, parasternal long-axis view, apical four-chamber view, and apical two-chamber views, where the frame rate was set between 65 and 75 frames per second. In addition, foreshortening of the left atrium was avoided [17]. Briefly, echocardiographic parameters were averaged from a total of five cardiac cycles [12], all of which contained clear image quality. TTE was measured using a Vivid E9 ultrasound instrument (GE Healthcare, Wauwatosa, WI, USA), containing a sector transducer (2.5–3.75 MHz). The left atrial anteroposterior diameter was acquired at its greatest dimension for parasternal long-axis view. The LV end-diastolic diameter (LVEDd) and LV end-systolic diameter (LVESd) were obtained at the level of the mitral valve tips using the M-mode Doppler ultrasonography. Left ventricle ejection fraction (LVEF) was automatically calculated by the system. The biplane modified Simpson's method of discs was used to separately calculate the left ventricular end-diastolic volume (LVEDV), left ventricular end-systolic volume (LVESV), maximum LA volume (LAVmax), and LA minimum volume (LAVmin) from apical 4- and 2-chamber views. The LAEF was calculated using the following formula: (LAVmax − LAVmin)/LAVmax × 100%. The left atrial transverse and longitudinal diameters were measured in a 4-chamber view of the end-ventricular systole. A spectral Doppler tissue imaging was used to measure tissue Doppler velocities at the septal and lateral annuli while pulsed Doppler echocardiography was used to record flow velocities of the valves. The ratio of the peak early mitral annular velocity to *E* wave (*E*/*e*′) was calculated and recorded.

### 2.2. Transesophageal Echocardiography

**Figure 2:**
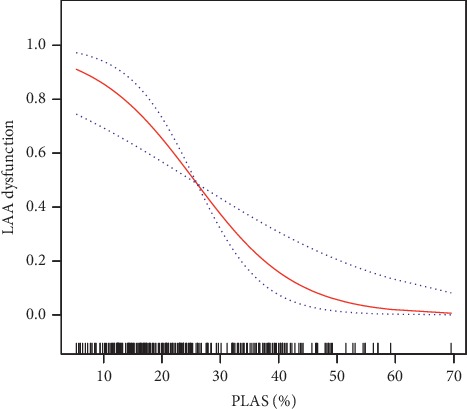
A linear relationship between PLAS and LAA dysfunction was observed. There was no inflection point.

During TEE examination (Figure 2), images were acquired using a transesophageal probe (2.9–7.0 MHz). Briefly, LAAeV was measured using a pulsed wave Doppler, where the sample volume was placed 1 cm distal from the mouth of the appendage. The maximum emptying velocity was calculated from an average of five well-defined emptying waves [18]. The LAT was defined as a fixed or mobile echogenic mass that clearly differed from the LA or LAA tissue. SEC was defined when dynamic, “smoke-like” echoes were seen within the atria and could not be eliminated by altering the gain settings [19, 20]. According to TEE findings, (1) the LAAeV < 40 cm/s and (2) LAT/SEC was found regardless of LAAeV; if either one is positive, the condition of LAA dysfunction was met [7].

### 2.3. Speckle Tracking Imaging

Cine loops were used to determine speckle tracking analysis (Figure 2) At least 5 consecutive beats from the three standard apical views were collected for analyses. Average PLAS was measured at the end of the reservoir phase with the tracking location marker placed at the end of the QRS wave calculated from 5 beats in patients with AF rhythm [21]. The LA endocardial border was manually traced in both the four- and two-chamber views with the regions of interest (ROI) encompassing the endocardial border of the mitral annulus, carefully excluding pulmonary veins and the LA appendage orifices. The thickness of the ROI was adjusted to the thinnest part to adapt to the atria. The data were analyzed using EchoPAC software (version 201) by two independent echocardiologists, who were blinded to clinical as well as other identifiers revealing patient characteristics.

### 2.4. Statistical Analysis

Data were analyzed using R (http://www.R-project.org) and Empower Stats software (http://www.empower.stats.com, X&Y solutions, Inc., Boston, MA, USA). Continuous variables were analyzed using the *t*-test (normal distribution) or Kruskal–Wallis rank sum test (nonnormal distribution), and categorical variables were analyzed using the *χ*^2^ test. Variables associated with LAAeV were evaluated by univariate logistics regression analysis, and those with a *p* value <0.1 were further analyzed using the multivariable logistics regression model to test for independence. The receiver operating characteristic (ROC) curve was used to evaluate the prognostic predictive value of PLAS in LAA dysfunction. Two-sided *p* values with *p* < 0.05 are considered statistically significant.

Reproducibility of the PLAS measurement was assessed in 20 randomly selected subjects. The inter- and intraobserver correlation coefficients were 0.8 and 0.9, respectively, which are consistent with those of previous work [22].

## 3. Results

### 3.1. Patient Characteristics

The basic characteristics of patients used in this study are depicted by a flow diagram (Figure 1). On the basis of inclusion and exclusion criteria, a total of 249 patients undergoing both TEE and TTE were included in the study. The mean age of the patients was 59.7 years (ranging from 49.7 to 69.7 years), and the majority of the patients were male (72.7%). Following TEE measurement, it was observed that LAA dysfunction was present in 120 patients, with an incidence rate of 48.2%, where 118 patients exhibited LAAeV < 40 cm/s. Out of the 120 identified patients with the LAA dysfunction, 43 had LAT/SEC and 41 showed a LAAeV < 40 cm/s measurement. The basic characteristics of patients with and without LAA dysfunction are presented in Table 1. Patients with LAA dysfunction were older (61.4 ± 9.7 vs. 58.1 ± 12.0, *p*= 0.021) and had a higher heart rate (83.6 ± 24.2 vs. 76.7 ± 22.9, *p*= 0.006) compared to controls. The number of patients presenting both LAA dysfunction and AF rhythm (OR 3.99, 95% CI: 2.36, 6.76, *p* < 0.0001) were statistically significant.

### 3.2. Univariate and Multiple Logistic Regression Analysis of LAA Dysfunction

**Figure 3:**
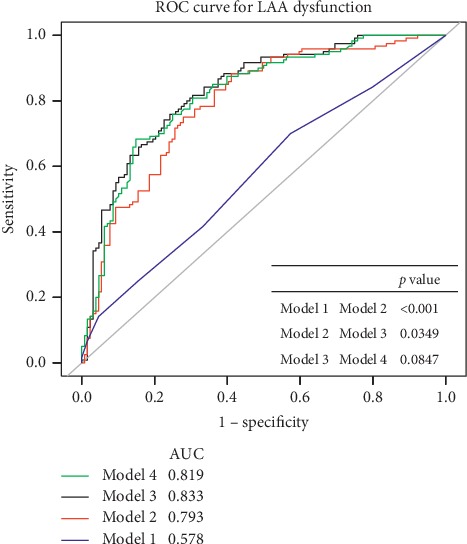
Comparisons among the four models used to diagnose LAA dysfunction using the ROC curve. Only the CHA2DS2-VASc score showed the lowest AUC value. CHA2DS2-VASc score, LAEF, and PLAS combined showed the highest AUC value. PLAS demonstrated similar accuracy and discriminability with Model 3. Model 1, CHA2DS2-VASc score; Model 2, CHA2DS2-VASc score + LAEF; Model 3, CHA2DS2-VASc score + LAEF + PLAS; Model 4, PLAS.

Univariate analysis showed that 18 variables, including age(OR 1.03, 95% CI: 1.00, 1.05, *p*=0.0224), heart rhythm (OR 3.99, 95% CI: 2.36, 6.76, *p* < 0.0001), heart rate (OR 1.01, 95% CI: 1.00, 1.02, *p*=0.0243), CHA2DS2-VASc score (OR 1.22, 95% CI: 1.04, 1.42, *p*=0.0141), LAD (OR 1.20, 95% CI: 1.13, 1.27, *p* < 0.0001), LA longitudinal diameter (OR 1.16, 95% CI: 1.11, 1.22, *p* < 0.0001), LA transverse diameter (OR 1.17, 95% CI: 1.11, 1.23, *p* < 0.0001), LAVmax (OR 1.06, 95% CI: 1.04, 1.08, *p* < 0.0001), LAVmin (OR 1.09, 95% CI: 1.07, 1.12, *p* < 0.0001), LAEF (OR 0.92, 95% CI: 0.89, 0.94, *p* < 0.0001), *E*/*e*′ (OR 1.09, 95% CI: 1.02, 1.16, *p*=0.0127), LVDd (OR 1.18, 95% CI: 1.10, 1.25, *p* < 0.0001), LVSd (OR 1.17, 95% CI: 1.10, 1.25, *p* < 0.0001), LVDV (OR 1.03, 95% CI: 1.02, 1.05, *p* < 0.0001), LVSV (OR 1.05, 95% CI: 1.03, 1.08, *p* < 0.0001), Simpson LVEF (OR 0.95, 95% CI: 0.92, 0.98, *p*=0.0043), and PLAS (OR 0.89, 95% CI: 0.86, 0.92, *p* < 0.0001) were associated with LAA dysfunction (Table 2). These identified variables were then further analyzed using multiple logistic regression, and PLAS (OR 0.90, 95% CI: 0.85, 0.95, *p* < 0.0005) was the only parameter significantly associated with LAA dysfunction after adjusting for sex, PMH of ablation, International Normalized Ratio (INR), AF, and isovolumic relaxation time (IVRT) (Table 2). The multivariate regression analysis showed that LAA dysfunction was consistently related to the tertials of the PLAS (Table 3). There was a linear relationship between PLAS and LAA dysfunction (Figure 3).

### 3.3. Sensitivity Analysis

**Figure 4:**
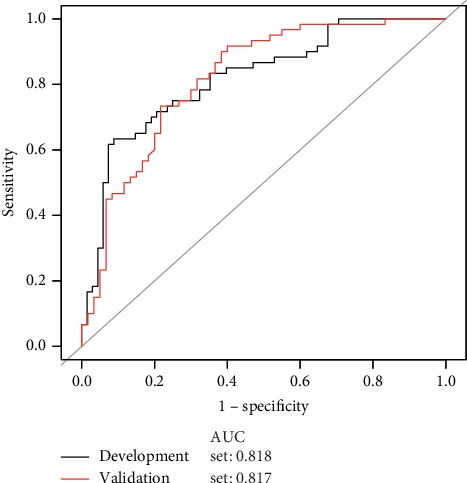
A receiver operating characteristic curve of PLAS that was used to predict the LAA dysfunction. The receiver operating characteristic curve of PLAS predicting the LAA dysfunction, having 50% of cases as modeling data and 50% of cases as validation data; the AUC were 0.818 and 0.817. The model showed a good discrimination ability on the validation datasets.

The ROC curve for the predictive models of LAA dysfunction are presented in Figure 3. Model 1 includes the CHA2DS2-VASc score only, while Model 2 includes both the CHA2DS2-VASc score and LAEF. Model 3 includes all the three factors, viz., CHA2DS2-VASc score, LAEF, and PLAS, while Model 4 includes PLAS only. Different models performances were compared, and it was observed that PLAS of Model 4 demonstrated similar accuracy and discriminability with Model 3, including CHA2DS2-VASc score, LAEF, and PLAS. For analyses, all subjects were categorized into two equal groups, viz., development and validation groups (Figure 4). The AUC for the development group was 0.818 (95% CI: 0.744, 0.818), yielding a sensitivity of 63.33% with a specificity of 91.18% at the optimal cutoff value (PLAS = 20.44%). In the validation set, the AUC was 0.817 (95% CI: 0.741, 0.894), along with a sensitivity of 78.33% and a specificity of 78.33% at the corresponding threshold.

## 4. Discussion

In this study, we performed a cross-sectional study of 249 participants to investigate the association between the functions of LA and LAA. Our results revealed a positive correlation between the reduced peak LA longitudinal strain, assessed by STE, and the presence of LAA reduced emptying velocity and/or thrombus on TEE examination in AF patients. Multivariate logistic regression analysis showed that a decrease in PLAS was an independent predictor of LAA dysfunction. By comparing the different models, it was observed that PLAS alone showed strong discriminability. These results indicate that in real life settings, PLAS can alternatively be used to rule out the possibility of LAA thrombus and to determine subsequent needs for other imaging assessments.

CHA2DS2-VASc scores, including risk factors of age, hypertension, heart failure, and diabetes mellitus, are clinically measurable indicators promoting atrial remodeling and are widely used to predict the risk of stroke in patients with AF. In our study, we did not find the CHA2DS2-VASc score to be significantly associated with LAA dysfunction, and this score was not an ideal platform to predict risks associated with stroke using the LAT value as a comparator. This inconsistency was also observed in previous studies [23, 24]. It is clear that the scoring system includes an assessment of the risks of early stroke, including those that cause vascular wall injury and hypercoagulable status [25, 26]. However, the scoring system did not analyze the effect of AF itself on cardiac remodeling which is a continuous present risk factor for stroke. Other possible variations include AF duration, structural cardiac abnormalities, race, anticoagulation adequacy, and other cardiovascular risk factors that might be associated with the observed effects. Yet, these indicators are relatively difficult to define. Consequently, recent efforts are focused on studies to refine the assessment of the risk of stroke based on cardiac structure and function [27, 28].

AF has been associated with electromechanical remodeling of the LA. LAA plays a role in maintaining LA pressure as an actively contracting structure [29]. As result of the intravascular volume status and remodeling in AF, LAA has become the primary location for the formation of thrombus [30, 31]. In the current study, we observed LAA as the site of thrombi in all patients. TEE is considered as the gold standard for the assessment of the LAA function [32]. However, as it is a semi-invasive procedure not tolerated by all patients, it remains controversial as to whether it is rational to use it as a measurement tool for the serial resolution of LAT or SEC in patients with a history of LAT or SEC [33]. Direct assessments of LAA function by TTE have also been proposed. Conventional 2D TTE has a low sensitivity of only 3–19% for the detection of thrombi in LA and especially in LAA [34]. Studies, such as those performed by Tamura et al. [35] as well as Uretsky et al. [36], were carried out to resolve the problem. These studies found that TTE-measured LAA tip Doppler tissue velocity was an independent predictor of LAA thrombus formation and may be a useful indicator for risk stratification of AF. Nevertheless, LAA is generally difficult to be clearly and totally visualized on TTE, especially in patients with small LAA.

Kuppahally et al. [13] showed that the LA strain inversely correlates with LA fibrosis in AF patients, suggesting that fibrosis decreases with LA compliance. Replacement of healthy atrial tissue with fibrotic tissue in AF leads to a reduction in the atrial contractile function and blood stasis, which results in the process of thrombus formation [37–39]. Our study observed that an impaired PLAS independently correlates with a decreased LAA function. It was also found to be the strongest independent predictor of LAA dysfunction multivariate analysis. Other conventional TTE parameters were found to be significant in univariate analysis but no longer maintained their significance in multivariate analysis. The associations between LA strain and TEE changes were also described in a previous study performed by Meng-Ruo Zhu et al. [40]. The difference here is that we defined LAA dysfunction as both decreased LAAeV and presence of LAT/SEC considering patient PMH may reduce the incidence of LAT/SEC. Therefore, we had a higher LA strain threshold for predicting LAA dysfunction compared to the one used in other studies [40]. Furthermore the diagnostic performance of PLAS to detect LAA dysfunction is strong. The addition of the CHA2DS2-VASc score and LAEF to PLAS offered no further discrimination in the detection of LAA dysfunction to that offered by PLAS alone, which suggests that in real-world practice, when an AF patient cannot undergo TEE, the PLAS can alternatively be used to rule out the possibility of thrombus and to determine the subsequent needs for other imaging assessments.

Our study has few limitations including the fact that it is composed of a small sample size with infrequent LAT events, which impaired our ability to analyze many covariates within a single regression model. Thus, we meticulously chose the covariates to be analyzed in the logistic regression models. It is still possible that some weakly associated parameters may have been missed. Secondly, the PLAS was calculated using EchoPAC software (version 201). Vendor differences should be considered when using different machines. Finally, we used the current software for LV analysis to study the LA pattern strain because a dedicated software for LA analysis has not yet been released.

Thus, it can be concluded that a decrease in PLAS is significantly associated with LAA dysfunction in patients with nonvalvular AF, providing a parameter to identify patients with nonvalvular AF at high risk of stroke.

## Figures and Tables

**Table 1:** Clinical characteristics of patients involved in this study.

	All patients	LAA dysfunction	Normal LAA function	*p* value
	(*n* = 249)	(*n* = 120)	(*n* = 129)	
Age (years)	59.7 ± 11.0	61.4 ± 9.7	58.1 ± 12.0	0.021
Gender (M/F)	181/68	92/28	89/40	0.174
Course of AF, months	43.6 ± 58.0	46.8 ± 62.2	40.5 ± 53.7	0.391
History of AF ablation	30 (12.0%)	15/105	15/114	0.833
INR				0.352
<2	217 (91.6%)	103 (89.6%)	114 (93.4%)	
≥2	20 (8.4%)	12 (10.4%)	8 (6.6%)	
Heart rhythm				<0.001
Atrial ﬁbrillation, *n* (%)	128 (51.4%)	79 (65.8%)	42 (32.6%)	
Sinus rhythm, *n* (%)	121 (48.6%)	41 (34.2%)	87v(67.4%)	
Heart rate (bpm)	80.0 ± 23.7	83.6 ± 24.2	76.7 ± 22.9	0.006
Hypertension, *n* (%)	111 (44.6%)	55 (45.8%)	56 (43.4%)	0.701
Diabetes mellitus, *n* (%)	43 (17.3%)	22 (18.3%)	21 (16.3%)	0.668
Myocardiopathy, *n* (%)	6 (2.4%)	3 (2.5%)	3 (2.3%)	1.000
Coronary heart disease, *n* (%)	35 (14.1%)	16 (13.3%)	19 (14.7%)	0.752
CHA2DS2-VASc score	2.2 ± 1.7	2.4 ± 1.8	1.9 ± 1.5	0.031
Medications, *n* (%)				
Antiplatelet drugs	28 (11.2%)	16 (13.3%)	12 (9.3%)	0.314
Anticoagulation drugs	77 (30.9%)	49 (40.8%)	28 (21.7%)	0.001
Antihypertension drugs	116 (46.6%)	59 (49.2%)	57 (44.2%)	0.431
Hypoglycemic agent	32 (12.9%)	16 (13.3%)	16 (12.4%)	0.827
Echocardiography				
LAAeV, *n* (%)				<0.001
<40 cm/s	118 (47.4%)	118 (98.3%)	0 (0.0%)	
≥40 cm/s	131 (52.6%)	2 (1.7%)	129 (100.0%)	
LAT/SEC, *n* (%)				<0.001
Yes	43 (17.3%)	43 (35.8%)	0 (0.0%)	
No	206 (82.7%)	77 (64.2%)	129 (100.0%)	
LAD (mm)	39.4 ± 5.9	41.9 ± 4.7	37.0 ± 6.0	<0.001
LA transverse diameter (mm)	39.5 ± 6.1	42.0 ± 5.7	37.3 ± 5.5	<0.001
LA longitudinal diameter (mm)	48.0 ± 7.2	51.2 ± 6.6	45.0 ± 6.4	<0.001
LAVmax (ml)	50.5 ± 21.7	60.2 ± 22.9	41.6 ± 16.1	<0.001
LAVmin (ml)	31.5 ± 18.6	41.0 ± 19.7	22.8 ± 12.2	<0.001
LAEF (%)	40.5 ± 14.5	33.30 ± 11.75	47.14 ± 13.55	<0.001
LVEDD (mm)	46.2 ± 4.6	47.8 ± 4.5	44.8 ± 4.3	<0.001
LVESD (mm)	29.7 ± 4.7	31.3 ± 5.1	28.2 ± 3.8	<0.001
LVEDV (ml)	58.2 ± 18.8	63.3 ± 19.5	53.4 ± 16.8	<0.001
LVESV (ml)	26.7 ± 12.8	30.6 ± 14.3	23.1 ± 10.0	<0.001
Simpson LVEF (%)	63.0 ± 7.9	61.5 ± 8.7	64.5 ± 6.9	0.003
IVRT (ms)	84.8 ± 24.8	84.1 ± 23.5	85.4 ± 26.1	0.695
*E*/*E*′	11.1 ± 4.3	11.9 ± 4.7	10.5 ± 3.7	0.010
Peak strain of LA	26.0 ± 13.2	18.6 ± 8.9	33.0 ± 12.9	<0.001

LAA dysfunction is defined as the left atrial appendage emptying velocity <40 cm/s or left atrial appendage with thrombi/spontaneous echocardiographic contrast. Data are expressed as mean ± SD, number (percentage) of subjects, or median (interquartile range). CI, confidence interval; AF, atrial ﬁbrillation; ACEI/ARB, angiotensin-converting enzyme inhibitors/angiotensin receptor blockers; LAT/SEC, left atrial thrombi/spontaneous echo contrast; LAD, left atrial dimension; LVDd, left ventricular end-diastolic dimension; LVSd, left ventricular end-systolic dimension; LVDV, left ventricular end-diastolic volume; LVSV, left ventricular end-systolic volume; LVEF, left ventricular ejection fraction; *E*/*E*′, the ratio of the early transmitral flow velocity to the early mitral annular velocity; IVRT, isovolumic relaxation time; LAVmax, left atrial maximum volume; LAVmin, left atrial minimum volume; LAEF, left atrial emptying fraction; LAAeV, left atrial appendage emptying ﬂow velocity.

**Table 2:** Comparison of the characteristics of patients with or without LAA dysfunction.

Covariate	Univariable			Multivariable		
	OR	95% CI	*p* value	OR	95% CI	*p* value
Sex						
Female	Ref.			—		
Male	1.48	0.84, 2.60	0.1756	—	—	—
PMH of ablation						
No	Ref.			—		
Yes	1.09	0.51, 2.33	0.8327	—	—	—
INR						
<2	Ref.			—		
≥2	1.66	0.65, 4.22	0.2871	—	—	—
Course of AF	1.00	1.00, 1.01	0.3895	—	—	—
Heart rhythm						
Sinus rhythm	Ref.			Ref.		
AF rhythm	3.99	2.36, 6.76	<0.0001	1.88	0.65, 5.42	0.2416
Age	1.03	1.00, 1.05	0.0224	1.02	0.97, 1.06	0.4670
Heart rate	1.01	1.00, 1.02	0.0243	0.99	0.97, 1.01	0.3481
CHA2DS2-VASc score	1.22	1.04, 1.42	0.0141	0.98	0.75, 1.28	0.9004
IVRT	1.00	0.99, 1.01	0.6937	—	—	—
LAD (mm)	1.20	1.13, 1.27	<0.0001	0.97	0.88, 1.05	0.4337
LA longitudinal diameter (mm)	1.16	1.11, 1.22	<0.0001	1.00	0.92, 1.08	0.9612
LA transverse diameter (mm)	1.17	1.11, 1.23	<0.0001	0.97	0.87, 1.08	0.5679
LAVmax (ml)	1.06	1.04, 1.08	<0.0001	0.98	0.86, 1.11	0.7110
LAVmin (ml)	1.09	1.07, 1.12	<0.0001	1.07	0.88, 1.29	0.4870
LAEF (%)	0.92	0.89, 0.94	<0.0001	1.00	0.91, 1.11	0.9871
*E*/*e*′	1.09	1.02, 1.16	0.0127	1.06	0.95, 1.17	0.2920
LVDd (mm)	1.18	1.10, 1.25	<0.0001	1.18	1.02, 1.36	0.0262
LVSd (mm)	1.17	1.10, 1.25	<0.0001	0.96	0.82, 1.13	0.6274
LVDV (ml)	1.03	1.02, 1.05	<0.0001	1.04	1.00, 1.08	0.0644
LVSV (ml)	1.05	1.03, 1.08	<0.0001	0.98	0.93, 1.04	0.4685
Simpson LVEF	0.95	0.92, 0.98	0.0043	1.00	0.93, 1.07	0.9652
Peak strain of LA	0.89	0.86, 0.92	<0.0001	0.90	0.85, 0.95	0.0005

CI, confidence interval; AF, atrial ﬁbrillation; ACEI/ARB, angiotensin-converting enzyme inhibitors/angiotensin receptor blockers; LAT/SEC, left atrial thrombi/spontaneous echo contrast; LAD, left atrial dimension; LVDd, left ventricular end-diastolic dimension; LVSd, left ventricular end-systolic dimension; LVDV, left ventricular end-diastolic volume; LVSV, left ventricular end-systolic volume; LVEF, left ventricular ejection fraction; *E*/*E*′, the ratio of the early transmitral flow velocity to the early mitral annular velocity; IVRT, isovolumic relaxation time; LAVmax, left atrial maximum volume; LAVmin, left atrial minimum volume; LAEF, left atrial emptying fraction; LAAeV, left atrial appendage emptying ﬂow velocity.

**Table 3:** The linearity between PLAS and LAA dysfunction.

Variables	Incidence, *n* (%)	Crude model			Multivariate-adjusted model 1			Multivariate-adjusted model 2		
		OR (95% CI)	*p* value	*p* for trend	OR (95% CI)	*p* value	*p* for trend	OR (95% CI)	*p* value	*p* for trend
PLAS (continuous, %)	Tertiles (*n* = 248)	0.90 (0.85, 0.95)	0.0005		0.87 (0.83, 0.92)	<0.0001		0.90 (0.85, 0.95)	0.0004	
T1: 5.3–17.3	82 (33.3)	1		<0.0001	1		<0.0001	1		0.0340
T2: 17.4–32.0	83 (33.3)	0.23 (0.11, 0.45)	<0.0001		0.23 (0.10, 0.53)	0.0007		0.58 (0.20, 1.65)	0.3046	
T3: 32.1–69.5	83 (33.3)	0.05 (0.02, 0.11)	<0.0001		0.04 (0.01, 0.14)	<0.0001		0.20 (0.04, 0.93)	0.0398	

Odds ratios were derived from the multivariate logistic regression analysis. Crude, no adjustment. Model I, adjusted for gender, PMH of ablation, INR, course of AF, age, heart rhythm, heart rate, and CHA2DS2-VASc score. Model II, adjusted for gender, PMH of ablation, INR, course of AF, age, heart rhythm, heart rate, CHA2DS2-VASc score, LAD, LA longitudinal diameter, LA transverse diameter, LAVmax, LAVmin, LAEF, *E*/*e*′, LVDd, LVSd, LVDV, LVSV, and Simpson LVEF. PLAS, peak left atrial strain. CI, confidence interval.

## Data Availability

The data used to support the findings of this study are available from the corresponding author upon request. The data are not publicly available due to the potential revealing of information compromising patient identity.

## Abbreviations

AF: Atrial fibrillation
IVRT: Isovolumic relaxation time
LA: Left atria
LAA: Left atrial appendage
LAAeV: Left atrial appendage emptying velocity
LAD: Left atrial dimension
LAEF: Left atrial emptying fraction
LAT: Left atrial thrombi
LAVmax: Left atrial maximum volume
LAVmin: Left atrial minimum volume
LVDd: Left ventricular end-diastolic dimension
LVDV: Left ventricular end-diastolic volume
LVEF: Left ventricular ejection fraction
LVSd: Left ventricular end-systolic dimension
LVSV: Left ventricular end-systolic volume
SEC: Spontaneous echocardiographic contrast
STE: Speckle tracking echocardiography
TEE: Transesophageal echocardiography
TTE: Transthoracic echocardiography
PLAS: Peak left atrial strain.
